# Study of the standard model with weak decays of charmed hadrons at BESIII

**DOI:** 10.1093/nsr/nwab181

**Published:** 2021-10-05

**Authors:** Hai-Bo Li, Xiao-Rui Lyu

**Affiliations:** Institute of High Energy Physics, Chinese Academy of Sciences, Beijing 100049, China; University of Chinese Academy of Sciences, Beijing 100049, China; University of Chinese Academy of Sciences, Beijing 100049, China

**Keywords:** charmed mesons, charmed baryon, leptonic decay, semi-leptonic decay, lepton flavor universality

## Abstract

A comprehensive review of weak decays of charmed hadrons (*D*^0/ +^, $D^+_s$ and $\Lambda ^+_c$) based on analyses of the threshold data from *e*^+^*e*^−^ annihilation in the BESIII experiment is presented. Current experimental challenges and successes in understanding decays of the charmed hadrons are discussed. Precise calibrations of quantum chromodynamics and tests of the standard model are provided by measurements of purely leptonic and semi-leptonic decays of charmed hadrons, and lepton universality is probed in purely leptonic decays of charmed mesons to three generations of leptons. Quantum correlations in threshold data samples provide access to strong phases in the neutral *D* meson decays and probe the decay dynamics of the charmed Λ_*c*_ baryon. Charm physics studies with near-threshold production of charmed particle pairs are unique to BESIII, and provide many important opportunities and challenges.

## Teaser

The BESIII experiment aims to unveil the fundamental physics inside the charmed hadrons, which consists of one charm quark and light quark(s). With unique advantage of threshold production and quantum coherence, BESIII has been very successful in examining the standard model and searching for new physics in particle physics.

## INTRODUCTION

The discovery of the *J*/ψ in 1974 marked a new era in particle physics. The arrival of the first heavy quark indicated that the standard model (SM) provided a correct low-energy description of particle physics. Four decades later, the charmed quark still plays unique roles in studies of strong and weak interactions [1]. Recent observation of *CP* violation in charmed meson decays has attracted significant and renewed interest to charm physics [2,3]. It paves the road to precise tests of the SM in interesting weak interaction transitions and maybe even to searches for new physics beyond the SM.

**Figure 1.**
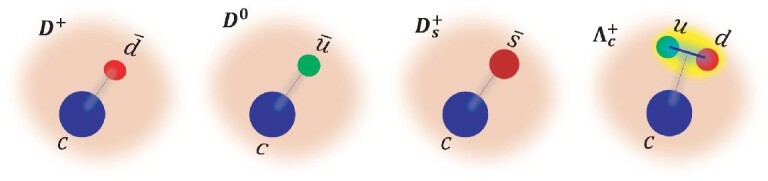
Quark constituents for the ground-state charmed hadrons of $D^+(c\bar{d}$), $D^0(c\bar{u}$), $D_s^+(c\bar{s}$) and $\Lambda _c^+(cud$). Taken from [6].

A distinctive feature of all the charmed hadrons is that their masses place them at the edge of the region where non-perturbative hadronic physics is operative, forcing us to develop new means to cope with such scales. This point has been made in prescient reviews [4,5] that posed many of the questions that are still awaiting answers. While this fact does not markedly affect the theoretical description of leptonic and semi-leptonic decays of charmed hadrons, it poses challenges to analyses of their hadronic transitions. We expect that detailed experimental studies would provide some hints on the dynamics of charm hadronic decays, so that eventually those problems will be overcome. In this review we focus on the weak decays of ground-state charmed hadrons, i.e. three charmed mesons $D^+(c\bar{d}$), $D^0(c\bar{u}$) and $D_s^+(c\bar{s}$) as well as one charmed baryon $\Lambda _c^+(cud$), with internal quark constituents as depicted in Fig. 1, that can be extensively studied using data collected at the BESIII experiment. There are mainly three classes of charmed hadron decays: purely leptonic, semi-leptonic and hadronic decays. Measurements of the charmed hadron decays can be used to calibrate lattice quantum chromodynamics (LQCD) calculations. In addition, BESIII data provide stringent constraints on the Cabibbo-Kobayashi-Maskawa (CKM) six-quark flavor-mixing matrix [7] via: (1) precision measurements of the CKM matrix elements |*V*_*cs*_| and |*V*_*cd*_| that parameterize the strengths of *c* → *s* and *c* → *d* weak transitions, respectively; (2) determinations of the strong-interaction phases in *D*-meson decays that are essential inputs to measurements of the *CP*-violating phase γ of the CKM matrix element *V*_*ub*_ in *B*-meson decays [8].

## ADVANTAGES NEAR THRESHOLD PRODUCTION FROM *e*^+^*e*^−^ ANNIHILATION

Experiments at *e*^+^*e*^−^ machines operating at the ψ(3770) and ψ(4140) resonances and $\Lambda ^+_c \bar{\Lambda }^-_c$ threshold, such as CLEO-c and BESIII [9], have several important advantages. First, the cross section for charm production is relatively high, for example, $\sigma (e^+e^- \rightarrow D^0\bar{D}^0) = (3.615\pm 0.010 \pm 0.038)$ nb and σ(*e*^+^*e*^−^ → *D*^+^*D*^−^) = (2.830 ± 0.011 ± 0.026) nb at the ψ(3770) peak [10]. Second, the $D\bar{D}$ and $\Lambda ^+_c \bar{\Lambda }^-_c$ pairs are produced in the exclusive two-body channel with no additional particles. Thus, one can employ a double-tag technique pioneered by the Mark III experiment [11]: a full reconstruction of an anti-*D* meson on one side of tagged events together with the known momentum and energy of colliding beams provides a ‘beam’ of *D* particles of known four-momentum on the other side. The tag yield, which provides the normalization for the branching fraction measurement, is extracted from the distribution of beam-constrained mass $M_\mathrm{BC}= \sqrt{E_\mathrm{beam}^2 - |\vec{p}_\mathrm{tag}|^2}$, where $\vec{p}_\mathrm{tag}$ is the three-momentum of the tag $\bar{D}$ candidate and *E*_beam_ is the beam energy, both evaluated in the *e*^+^*e*^−^ center-of-mass system. When a tagged *D*^+^ decays to a muon and a muonic neutrino, μ^+^ν_μ_, the mass of the (missing) nearly zero-mass neutrino can be inferred from energy-momentum conservation. This tagging technique, which obviates the need for knowledge of the luminosity or the production cross section, is a powerful tool for charmed particle decay measurements that is most accurately performed by the near-threshold experiments.

Furthermore, the charmed hadron pairs at BESIII are produced via *e*^+^*e*^−^ annihilation through a virtual photon (with spin, parity and *C* parity of *J*^*PC*^ = 1^−−^), e.g. in the process $e^+e^- \rightarrow \gamma ^* \rightarrow \psi (3770) \rightarrow D^0\bar{D}^0$ ($e^+e^- \rightarrow \gamma ^* \rightarrow \Lambda ^+_c \bar{\Lambda }^-_c$). Hence, the wave function of the produced charm hadron pairs is analogous to that of photons in an aligned, spin-1 state with odd charge parity *C* = −1, and the $D^0\bar{D}^0$ ($\Lambda ^+_c \bar{\Lambda }^-_c$) pair are in a quantum-entangled state. This allows for unique probes of the structure of decay amplitudes and relative phases between *D*^0^ and $\bar{D}^0$ decays, as well as novel measurements of neutral *D* mixing and *CP* violation in *D*^0^ and $\Lambda ^+_c$ decays [12–15].

## PRECISION TESTS OF THE STANDARD MODEL

In the SM, quark-flavor mixing is characterized by the unitary 3 × 3 CKM matrix [7]:

(1) \begin{eqnarray*} V_{\rm CKM} = \left(\begin{array}{@{}l@{\quad }l@{\quad}l@{}} V_{ud} & V_{us} & V_{ub} \\ V_{cd} & V_{cs} & V_{cb} \\ V_{td} & V_{ts} & V_{tb} \end{array}\right). \end{eqnarray*}

The CKM matrix induces flavor-changing transitions within and among generations in the charged currents in tree-level *W*^±^-exchange interactions. Experiments have revealed a strong hierarchy among the CKM matrix elements: transitions within the same generation are described by *V*_CKM_ elements of $\mathcal {O}(1)$, whereas there is a suppression of $\mathcal {O}(10^{-1})$ for transitions between the first and the second generations, $\mathcal {O}(10^{-2})$ between the second and the third, and $\mathcal {O}(10^{-3})$ between the first and the third. Following the observation of this hierarchy, Wolfenstein [16] proposed an expansion of the CKM matrix in terms of four parameters (which was further modified by Buras [17]), λ, *A*, $\bar{\rho }$ and $\bar{\eta }$, under the relations

(2) \begin{eqnarray*} \lambda ^2 &=&\frac{|V_{us}|^2}{|V_{ud}|^2+|V_{us}|^2},\nonumber\\ A^2\lambda ^4 &=&\frac{|V_{cb}|^2}{|V_{ud}|^2+|V_{us}|^2},\nonumber\\ \bar{\rho }+i\bar{\eta }&=& -\frac{V_{ud}V_{ub}^*}{V_{cd}V^*_{cb}}, \end{eqnarray*}

which are used to fully characterize the matrix. Any deviation of *V*_CKM_ from unitarity would indicate new physics beyond the SM. Therefore, improving our knowledge of the CKM matrix elements to test unitarity is one of the principal goals of flavor physics. BESIII data provide direct precise measurements of the CKM matrix elements |*V*_*cs*_| and |*V*_*cd*_| using the purely leptonic and semi-leptonic charmed-hadron decay rates, as discussed in detail below.

**Figure 2.**
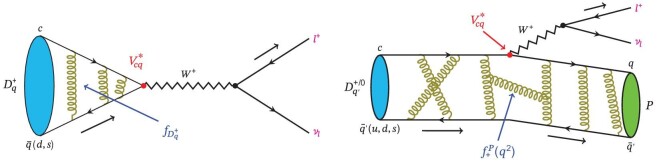
Diagrams for purely leptonic (left) and semi-leptonic (right) decays of *D*_(*s*)_ mesons. (Courtesy of Hao-Kai Sun, Institute of High Energy Physics, Chinese Academy of Sciences.)

Purely leptonic and semi-leptonic decays of hadrons have a special characteristic advantage in studies of the weak interaction [18,19]. A key feature is their relative simplicity, a consequence of the fact that in these processes the effects of the strong interactions can be isolated. For each decay type, the decay amplitude can be written as the product of a well-understood leptonic current for the process *W*^+^ → ℓ^+^ν_ℓ_ (ℓ denotes charged leptons) and a more complicated hadronic current for the quark transition. Figure 2 shows the Feynman diagrams for the purely leptonic (left diagram) and semi-leptonic (right diagram) decays. In purely leptonic decays, the hadronic current describes an annihilation of the quark and the anti-quark in the initial-state charmed mesons, while in semi-leptonic decays it describes the evolution from the initial-charmed hadron to the final-state hadrons. Because strong interactions affect only one of the two currents, purely leptonic and semi-leptonic decays are relatively simple from a theoretical perspective; they provide bilateral means both to measure fundamental SM parameters and to perform detailed studies of the decay dynamics [20].

### A bridge between quarks and leptons: decay constants and lepton flavor universality

Purely leptonic decays of the *D*^+^ and $D^+_s$ mesons are among the simplest and best-understood probes of *c* → *d* and *c* → *s* quark transitions. In each case, the effects of the strong interaction can be parameterized in terms of just one factor, called the decay constant $f_{D_{q}^+}$. In the SM, the corresponding decay rate, ignoring radiative corrections, is given in a simple form:

(3) \begin{eqnarray*} \Gamma (D_{q}^+ \rightarrow \ell ^+\nu _\ell )&=& \frac{G^2_F f^2_{D_{q}^+}}{8\pi } | V_{cq} |^2 \ m^2_\ell m_{D_{q}^+}\nonumber\\ &&\times\, \bigg(1- \frac{m^2_\ell }{m^2_{D_{q}^+}}\bigg )^{2}. \end{eqnarray*}

Here *q* = *d* or *s* quark and ℓ = *e*, μ or τ (electron, muon or tau lepton), and ν_ℓ_ stands for the neutrino with the corresponding lepton flavor. The $D^+_q$ mass ($m_{D_{q}^+}$), the mass of the charged lepton (*m*_ℓ_) and the Fermi coupling constant (*G*_*F*_) are all known to high precision [21]. Thus, the determination of $\Gamma (D_{q}^+ \rightarrow \ell ^+\nu _\ell )$ directly measures the product $f_{D_{q}^+} |V_{cq}|$ of the $D^+_q$ decay constant and the magnitude of the *c* → *q* CKM matrix element. One can then either extract |*V*_*cq*_| by using the predicted value of $f_{D_{q}^+}$, e.g. from LQCD [22], or obtain $f_{D_{q}^+}$ by using the experimentally measured |*V*_*cq*_| to test the LQCD prediction.

Since the purely leptonic decays of pseudoscalar mesons are helicity suppressed, their decay rates are proportional to the square of the charged lepton mass. According to Equation (3), the SM-expected relative decay widths for the τν_τ_, μν_μ_ and *e*ν_*e*_ modes are 2.67 : 1 : 2.35 × 10^−5^ for *D*^+^ and 9.75 : 1 : 2.35 × 10^−5^ for $D^+_s$ with negligible uncertainties. Therefore, the SM $D^+_q \rightarrow e^+\nu _e$ branching fractions are expected to be $\mathcal {B}_{e^+\nu _e} < 10^{-8}$ and not yet experimentally observable.

**Figure 3.**
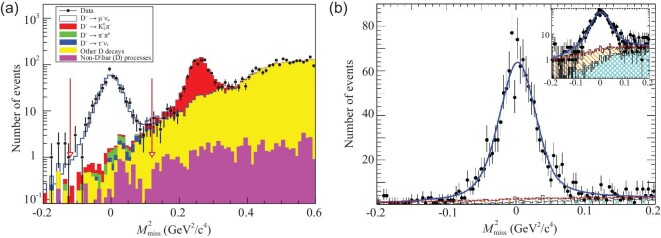
The missing-mass $M_\mathrm{miss}^2$ distribution of the selected (a) *D*^+^ → μ^+^ν_μ_ and (b) $D^{+}_s \rightarrow \mu ^{+} \nu _\mu$ candidates from [23] and [25], respectively. The error bars show the statistical uncertainty in experimental data. Arrows in plot (a) are the boundaries of the signal region, and the inset in plot (b) shows the same distribution on the logarithmic scale. Plots are from [23] and [25].

Using a data sample with an integrated luminosity of 2.93 fb^−1^ collected with BESIII at the ψ(3770) peak, a total of about 1.7 million single-tag *D*^−^ mesons are selected using nine hadronic decay modes (summing up to 30% of all *D*^−^ decays) on the tagging side. Throughout this article, charge-conjugate modes are implicitly assumed, unless otherwise stated. Signal candidates of *D*^+^ → μ^+^ν_μ_ are required to have a signature in which the tagging *D*^−^ mesons are accompanied by exactly one track that is identified as a muon with charge opposite to that of the tagging *D*^−^. Since the massless neutrino is undetected, the yields of the signal *D*^+^ → μ^+^ν_μ_ decays are measured based on the missing-mass-squared variable $M_\mathrm{miss}^2 = (E_\mathrm{beam}- E_{\mu })^2 - (-\vec{p}_\mathrm{tag}- \vec{p}_\mu )^2$, where *E*_μ_ and $\vec{p}_\mu$ are the energy and three-momentum of the muon, respectively, and $\vec{p}_\mathrm{tag}$ is the three-momentum of the tagged *D*^−^ candidate. Here *M*_miss_ corresponds to the invariant mass of the neutrino, and hence the signal for *D*^+^ → μ^+^ν_μ_ events is the peak around $M_\mathrm{miss}^2 = 0$, as shown in Fig. 3(a), where a tiny background is smoothly distributed under the signal peak. From this, BESIII obtained the world’s most accurate branching fraction measurement for *D*^+^ → μ^+^ν_μ_ decay [23], as shown in Table 1. By inputting either the LQCD-calculated value for the decay constant [22] or the CKM matrix element values from a global SM fit [21], the |*V*_*cd*_| or $f_{D^+}$ can be extracted; the corresponding results are listed in Table 1. Using the same data sample, BESIII recently reported the first measurement of the absolute decay branching fraction for *D*^+^ → τ^+^ν_τ_ with a significance of 5.1σ [24]. The presence of additional final-state neutrinos from the τ^+^ decays results in more background and a relatively larger systematic uncertainty than in the *D*^+^ → μ^+^ν_μ_ decay measurement.

**Table 1.** Measurements of *D*^+^ and $D^+_s$ purely leptonic decays with threshold data at BESIII, and comparisons between experimental results and theoretical expectation or SM-global fit results. (Here, ‘–’ indicates not available.)

Observable	Measurement	Prediction/fit
$\mathcal {B}(D^+\rightarrow \mu ^+\nu _\mu )$	(3.71 ± 0.19_stat_ ± 0.06_syst_) × 10^−4^ [23]	–
$f_{D^+}|V_{cd}|$	(45.75 ± 1.20_stat_ ± 0.39_syst_) MeV	–
$f_{D^+}$	(203.8 ± 5.2_stat_ ± 1.8_syst_) MeV	(212.7 ± 0.6) MeV [22]
|*V*_*cd*_|	0.2150 ± 0.0055_stat_ ± 0.0020_syst_	0.22438 ± 0.00044 [21]
$\mathcal {B}(D^+ \rightarrow \tau ^+ \nu _\tau )$	(1.20 ± 0.24_stat_ ± 0.12_syst_) × 10^−4^ [24]	–
Γ(*D*^+^ → τ^+^ν_τ_)/Γ(*D*^+^ → μ^+^ν_μ_)	3.21 ± 0.64_stat_ ± 0.43_syst_ [24]	2.67
$\mathcal {B}(D^+_s \rightarrow \mu ^+\nu _\mu )$	(5.49 ± 0.16_stat_ ± 0.15_syst_) × 10^−3^ [25]	–
$f_{D^+_s}|V_{cs}|$	(246.2 ± 3.6_stat_ ± 3.5_syst_) MeV	–
$f_{D^+_s}$	(252.9 ± 3.7_stat_ ± 3.6_syst_) MeV	(249.9 ± 0.5) MeV [22,26]
|*V*_*cs*_|	0.985 ± 0.014_stat_ ± 0.014_syst_	0.97359 ± 0.00011 [21]
$\Gamma (D^+_s \rightarrow \tau ^+ \nu _\tau )/ \Gamma (D^+_s \rightarrow \mu ^+ \nu _\mu )$	9.98 ± 0.52 [25]	9.74
$f_{D^+_s}/f_{D^+}$	1.24 ± 0.04_stat_ ± 0.02_syst_ [25]	1.1783 ± 0.0016 [28]

To study the $D^+_s \rightarrow \mu ^+ \nu _\mu$ signal channel, BESIII uses $e^+e^-\rightarrow D_s^+ D^{*-}_s$ collisions at the center-of-mass energy of 4178 MeV and performs a similar analysis as was used for the *D*^+^ → μ^+^ν_μ_ decay measurement [25]; the $D^+_s \rightarrow \mu ^+ \nu _\mu$ signal peak is shown in Fig. 3(b). The absolute branching fraction and the product $f_{D^+_s}|V_{cs}|$ are obtained as listed in Table 1. Taking the CKM matrix element |*V*_*cs*_| from the latest global SM fit [21], the $D^+_s$ decay constant is determined. Alternatively, taking the averaged decay constant from recent LQCD calculations [22,26], the CKM matrix element is extracted as listed in Table 1. These are the most precise measurements to date, and provide an important calibration of the theoretical calculations of $f_{D^+_s}$ and a stringent test of the unitarity of the CKM matrix with an improved accuracy.

Using the world average values from the Particle Data Group (PDG) [21], we determine the ratio

(4) \begin{eqnarray*} R^{D^+}_{\tau /\mu } &=& \Gamma (D^+ \rightarrow \tau ^+ \nu _\tau )/ \Gamma (D^+ \rightarrow \mu ^+ \nu _\mu )\nonumber\\ &=& 3.21 \pm 0.64_\mathrm{stat}\pm 0.43_\mathrm{syst}, \end{eqnarray*}

which, although still statistically limited, is consistent with the SM prediction of 2.67. With BESIII’s expected future 20 fb^−1^ data set at the ψ(3770) peak, as discussed in [9] and approved by the collaboration, the precision on $R^{D^+}_{\tau /\mu }$ will be statistically improved to about 8%, which will provide an important test of the lepton flavor universality (LFU). For the $D_s^+$, we obtain

(5) \begin{eqnarray*} R^{D^+_s}_{\tau /\mu } &=& \Gamma (D^+_s \rightarrow \tau ^+ \nu _\tau )/ \Gamma (D^+_s \rightarrow \mu ^+ \nu _\mu )\nonumber\\ &=& 9.98\pm 0.52, \end{eqnarray*}

which agrees with the SM-predicted value of 9.74. Meanwhile, $D^+_s \rightarrow \tau ^+ \nu _\tau$ decays are currently being studied at BESIII with an expected result that will have a precision comparable to that achieved for the $D^+_s \rightarrow \mu ^+ \nu _\mu$ decay mode. This result should improve the accuracy of the $f_{D^+_s}|V_{cs}|$ measurement and can also be used to test LFU in the ratio $R^{D^+_s}_{\tau /\mu }$ with a precision of 4.7% based on the current data set [9]. With the expected 6 fb^−1^ data set at 4178 MeV, as discussed in [9], the precision on $R^{D^+_s}_{\tau /\mu }$ will be systematically limited at about 3% or less, which will provide for the most stringent test of the μ–τ LFU in heavy quark decays [27].

Finally, combining the measured $f_{D^+_s}|V_{cs}|$ value with its $f_{D^+}|V_{cd}|$ counterpart, along with the |*V*_*cd*_/*V*_*cs*_| value from the global SM fit [21], BESIII made a direct measurement of the $f_{D^+_s}/f_{D^+}$ decay constant ratio [25] that is 1.5σ higher than the Flavour Lattice Averaging Group (FLAG) world average value [28], as shown in Table 1. Since LQCD can make a very accurate prediction of $f_{D^+_s}/f_{D^+}$, which is a unique property of purely leptonic $D^+/D^+_s$ decays, BESIII can make unambiguous measurements of fundamental SM parameters and perform detailed studies of the charmed hadron decay dynamics. For these purposes, more data are needed at the $D\bar{D}$ and $D_s^+ D_s^{*-}$ thresholds to pursue high-precision calibrations of LQCD calculations [9].

### Precision measurements of the transition form factors

In the SM, semi-leptonic decays of charmed hadrons involve the interaction of a leptonic current with a hadronic current. The latter is non-perturbative and cannot be calculated from first principles; thus, it is usually parameterized in terms of form factors. Still, the weak and strong effects in semi-leptonic decays can be well separated, since there are no strong final-state interactions between the leptonic and hadronic systems. Among the semi-leptonic decays, the simplest case is *D*^0/ +^ → *P*ℓ^+^ν_ℓ_ (where *P* denotes a pseudoscalar meson), for which the differential partial decay width is given, in the limit of negligible charged lepton mass, by

(6) \begin{equation*} \frac{{\mathrm{d}} \Gamma (D^{0/+} \rightarrow P \ell ^+ \nu _\ell )}{{\mathrm{d}}q^2}= \frac{G^2_F| V_{cq} |^2 }{24 \pi ^3} p^3_P | f^P_+(q^2) |^2 . \end{equation*}

Here *p*_*P*_ is the magnitude of the three-momentum of the *P* meson in the *D*^0/ +^ rest frame and $f^P_+(q^2)$ is the form factor of the hadronic weak current depending on *q*^2^ = |*M*(ℓ^+^ν_ℓ_)|^2^, the square of the four-momentum transfer between the initial state *D*^0/ +^ and final state *P*. Thus, semi-leptonic decays can be used to extract the product of a form factor normalization chosen to be at *q*^2^ = 0 and a CKM matrix element: $|V_{cq}| f^P_+ (0)$. These decays allow for a robust determination of the |*V*_*cs*_| and |*V*_*cd*_| CKM matrix elements in conjunction with form factors determined from LQCD calculations. Alternatively, by inputting CKM matrix elements one can determine the form factors to provide high-precision tests of LQCD calculations.

The CLEO experiment had made precision measurements of semi-leptonic charm-decay rates using a data set accumulated at the ψ(3770) peak [29]. With a three-times-larger data set, BESIII reported improved measurements of the absolute decay rates and the form factors, thereby assuming an important role in the precision tests of LQCD calculations [9].

**Figure 4.**
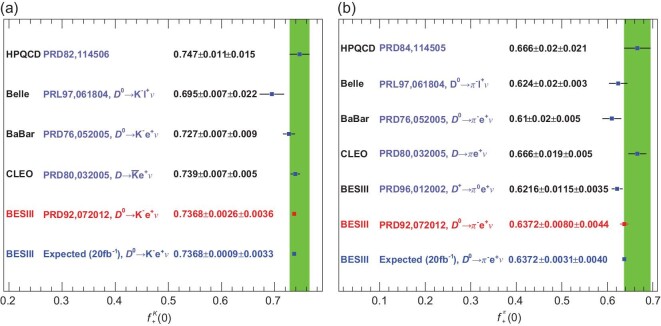
Comparison of the results for (a) $f^K_+(0)$ and (b) $f^\pi _+(0)$ measured by the Belle, BaBar, CLEO-c and BESIII experiments. The green bands present the LQCD uncertainties. The value marked in red denotes the best measurement from BESIII, and the value marked in dark blue denotes the expected precision from BESIII with ten times the current data set [9]. (Courtesy of Hai-Long Ma, Institute of High Energy Physics, Chinese Academy of Sciences.)

Notably, measurements of the exclusive *D*^0^ → *K*^−^ℓ^+^ν_ℓ_ and π^−^ℓ^+^ν_ℓ_ decays, as well as $D^+ \rightarrow \bar{K}^0 \ell ^+\nu _\ell$ and π^0^ℓ^+^ν_ℓ_ decay modes, with ℓ = *e* or μ, have been reported [30,31]. Results for the absolute branching fractions are summarized in Table 2. From studies of the differential decay rates (see Equation (6)), the products of the hadronic form factor at *q*^2^ = 0 and the magnitude of the CKM matrix element, $|V_{cs}| f^K_+ (0)$ and $|V_{cd}| f^\pi _+ (0)$, are shown in Table 2. Combining these products with the values of |*V*_*cs*_| and |*V*_*cd*_| from the SM-constrained fit [21], we extract the transition form factors

$$\begin{eqnarray*}
f^K_+ (0) = 0.7368 \pm 0.0026_\mathrm{stat}\pm 0.0036_\mathrm{syst}
\end{eqnarray*}$$

and

$$\begin{eqnarray*}
f^\pi _+ (0) =0.6372 \pm 0.0080_\mathrm{stat} \pm 0.0044_\mathrm{syst},
\end{eqnarray*}$$

and their ratio

$$\begin{eqnarray*}
\frac{f^\pi _+ (0)}{f^K_+ (0)} = 0.865\pm 0.013 ,
\end{eqnarray*}$$

which is in good agreement with the present average (0.834 ± 0.023) of LQCD calculations [27,28] and a light cone sum rule value 0.84 ± 0.04 [32]. The experimental precision is better than that of the theoretical calculation. The measurement of $f^\pi _+ (0)$ is dominated by statistical uncertainties. More data will reduce these uncertainties as discussed in the BESIII future physics programme [9]. Figure 4 shows the form factors $f^K_+ (0)$ and $f^\pi _+ (0)$ measured by various experiments together with results from LQCD calculations [27,28].

**Table 2.** Measurements of *D*^0^/*D*^+^, $D^+_s$ and Λ_*c*_ semi-leptonic decays with near-threshold data at BESIII, and comparisons between experimental results and theoretical expectation or SM-global fit results. (Here ‘–’ indicates not available.)

Observable	Measurement	Prediction/fit
$\mathcal {B}(D^0 \rightarrow K^- e^+ \nu _e)$	$(3.505 \pm 0.014_\mathrm{stat}\pm 0.033_\mathrm{syst})\%$ [30]	–
$|V_{cs}| f^K_+ (0)$	0.7172 ± 0.0025_stat_ ± 0.0035_syst_ [30]	–
$f^K_+ (0)$	0.7368 ± 0.0026_stat_ ± 0.0036_syst_ [30]	0.747 ± 0.011 ± 0.015 [28]
$\mathcal {B}(D^0 \rightarrow \pi ^- e^+ \nu _e)$	$(0.295 \pm 0.004_\mathrm{stat}\pm 0.003_\mathrm{syst})\%$ [30]	–
$|V_{cd}| f^\pi _+ (0)$	0.1435 ± 0.0018_stat_ ± 0.0009_syst_ [30]	–
$f^\pi _+ (0)$	0.6372 ± 0.0080_stat_ ± 0.0044_syst_ [30]	0.66 ± 0.02 ± 0.02 [28]
$\mathcal {B}(D^+ \rightarrow \bar{K}^0 e^+\nu _e)$	$(8.60 \pm 0.06_\mathrm{stat} \pm 0.15_\mathrm{syst})\%$ [31]	–
$f^K_+ (0)$	0.725 ± 0.004_stat_ ± 0.012_syst_ [31]	0.747 ± 0.011 ± 0.015 [28]
$\mathcal {B}(D^+ \rightarrow \pi ^0 e^+\nu _e )$	$(0.363\pm 0.008_\mathrm{stat} \pm 0.005_\mathrm{syst} )\%$ [31]	–
$f^\pi _+ (0)$	0.622 ± 0.012_stat_ ± 0.003_syst_ [31]	0.66 ± 0.02 ± 0.02 [28]
$f^\pi _+ (0)/f^K_+ (0)$	0.865 ± 0.013 [31]	0.84 ± 0.04 [32]
$\mathcal {B}(\Lambda ^+_c \rightarrow \Lambda e^+ \nu _e )$	$(3.63 \pm 0.38_\mathrm{stat} \pm 0.20_\mathrm{syst})\%$ [33]	–
$\mathcal {B}(\Lambda ^+_c \rightarrow \Lambda \mu ^+ \nu _\mu )$	$(3.49\pm 0.46_\mathrm{stat} \pm 0.27_\mathrm{syst})\%$ [34]	–
$\mathcal {B}(\Lambda ^+_c \rightarrow \Lambda \mu ^+ \nu _\mu )/ \mathcal {B}(\Lambda ^+_c \rightarrow \Lambda e^+ \nu _e)$	0.96 ± 0.16_stat_ ± 0.04_syst_ [34]	≈1.0

**Figure 5.**
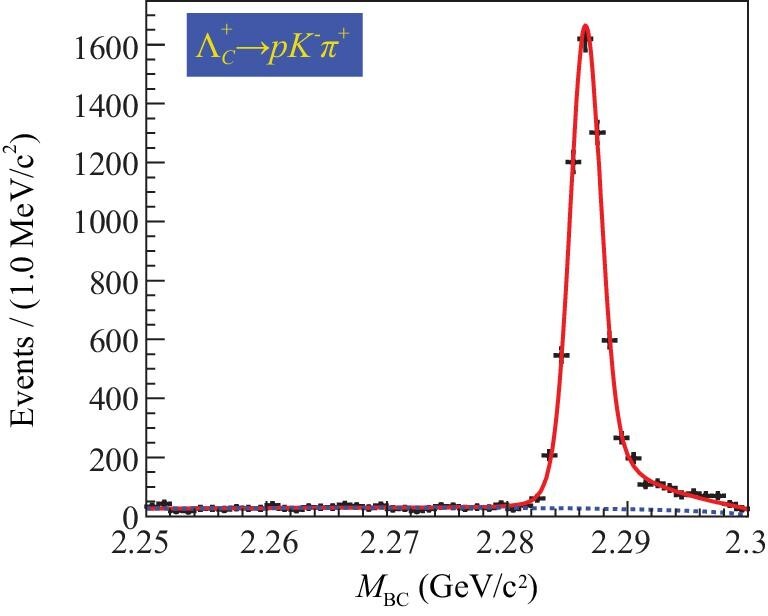
Fit to the *M*_BC_ distribution for $\bar{\Lambda }_c^- \rightarrow \bar{p} K^+ \pi ^-$ decay in the tag side. The points with error bars are data, the solid curves show the total fits and the dashed curves are the background shapes. (Courtesy of Pei-Rong Li, Lanzhou University.)

**Figure 6.**
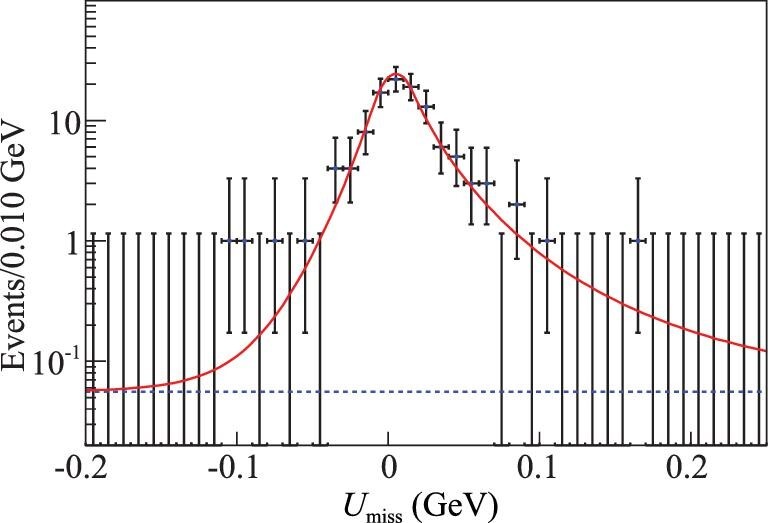
Fit to the *U*_miss_ distribution within the Λ signal region [33]. The points with error bars are data, the solid curves show the total fits and the dashed curves are the background shapes. Plot is from [33].

Based on a 567 pb^−1^ data set collected at 4.6 GeV, an energy point slightly above the $\Lambda ^+_c \bar{\Lambda }^-_c$ production threshold, BESIII made the first absolute branching fraction measurement of $\Lambda ^+_c \rightarrow \Lambda e^+ \nu _e$ [33]. Similar to the tagging technique employed in the $D\bar{D}$ threshold production, the $\bar{\Lambda }_c^{-}$ is tagged via its hadronic decay modes. As an example, Fig. 5 shows the beam-constrained mass *M*_BC_ distribution for the $\bar{\Lambda }_c^- \rightarrow \bar{p} K^+ \pi ^-$ tagging mode, where the background level is very low. This is typical for most tagging modes and demonstrates that the threshold data sets provide unique opportunities for nearly background-free charmed baryon decay measurements. Since the massless neutrino is undetected, the kinematic variable $U_{\text{miss}}= E_{\text{miss}}- c|\vec{p}_{\text{miss}}|$ is used to infer its presence, where *E*_miss_ and $\vec{p}_{\text{miss}}$ are the missing energy and missing momentum carried by the neutrino, respectively. The calculation methods of *E*_miss_ and $\vec{p}_{\text{miss}}$ can be found in [33]. The *U*_miss_ distribution is presented in Fig. 6, and a tiny background under the signal peak is inferred. From this, the absolute branching fractions for $\Lambda ^+_c \rightarrow \Lambda e^+ \nu _e$ and $\Lambda ^+_c \rightarrow \Lambda \mu ^+ \nu _\mu$ decays are determined. For the $\Lambda ^+_c \rightarrow \Lambda e^+ \nu _e$ case, the BESIII result listed in Table 2 corresponds to a two-fold improvement in the precision of the world average value. Since the branching fraction for $\Lambda ^+_c \rightarrow \Lambda e^+ \nu _e$ is the benchmark and serves as a normalization mode for all other $\Lambda _c^+$ semi-leptonic channels, the BESIII result allows for stringent tests of different theoretical models. For the muonic decay $\Lambda ^+_c \rightarrow \Lambda \mu ^+ \nu _\mu$, the BESIII result is the first direct measurement [34], and with it the branching fraction ratio is determined to be

$$\begin{eqnarray*}
&&\mathcal {B}(\Lambda ^+_c \rightarrow \Lambda \mu ^+ \nu _\mu )/ \mathcal {B}(\Lambda ^+_c \rightarrow \Lambda e^+ \nu _e)\nonumber\\
&&\qquad = 0.96\pm 0.16_\mathrm{stat} \pm 0.04_\mathrm{syst},
\end{eqnarray*}$$

which is consistent with *e* − μ LFU. The form factors for charmed baryon transition to light hyperons/baryons will be studied with high precision when more threshold data samples are collected by BESIII [9]. The detailed *q*^2^-dependent transition form factors can be studied at BESIII, and will provide unique calibrations of LQCD calculations. Alternatively, with LQCD predictions as input, BESIII measurements can be used to test LFU at any given *q*^2^ value.

### Impact on CKM matrix elements: |*V*_*cs*_| and |*V*_*cd*_|

**Figure 7.**
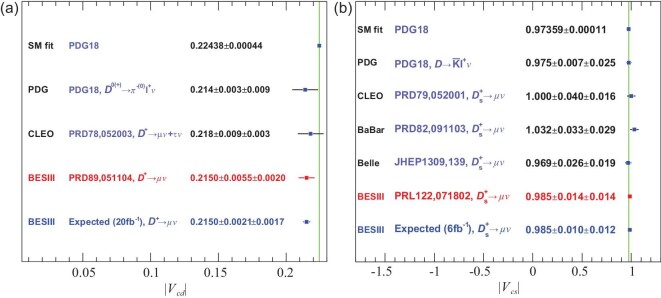
Precision of the measurements of (a) |*V*_*cd*_| and (b) |*V*_*cs*_|. The green bands indicate the uncertainties of the average values from the global fit in the SM [35]. The circles, dots and rectangles with error bars are results derived from semi-leptonic *D* decays, purely leptonic *D* decays and other methods, respectively. The results marked in red denote the best measurements, and the values marked in light blue denote the expected precisions with the BESIII data sets that will be accumulated in the future [9]. (Courtesy of Hai-Long Ma, Institute of High Energy Physics, Chinese Academy of Sciences.)

If precision LQCD calculations of the decay constants and form factors are taken as inputs, measurements of branching fractions for the purely leptonic and semi-leptonic decays can be used to confront weak-interaction physics. In the past decade, great progress has been made in the LQCD calculations of decay constants. The uncertainties of the results have been reduced from the level of 1%–2% to 0.2% [28]. With these, the BESIII leptonic-decay measurements have uncertainties of 2.5% and 1.5% for |*V*_*cd*_| and |*V*_*cs*_|, respectively, and dominate the PDG world average values. For leptonic decays, the statistical error on |*V*_*cd*_| is larger than the systematic error, while the statistical and systematic uncertainties of |*V*_*cs*_| are comparable, as shown in Fig. 7. The BESIII result for |*V*_*cd*_| listed in Fig. 7(a) is within 1.7σ of the value obtained from a global SM fit to the other CKM matrix element measurements that assumes unitarity.

With additional data from the next 10-year physics programme for BESIII [9], the relative errors on the |*V*_*cs*_| and |*V*_*cd*_| determinations with purely leptonic decays will both reach the 1% level; if the |*V*_*cd*_| result is the same as its current central value, the significance of the discrepancy would increase to about the 4σ level, as shown in Fig. 7(a).

In addition, with the FLAG [28] world average value for $(f_{D^+_s}/f_{D^+})^\mathrm{FLAG} = 1.1783 \pm 0.0016$, BESIII obtained |*V*_*cd*_/*V*_*cs*_|^2^ = 0.048 ± 0.003_stat_ ± 0.001_syst_, which is consistent with that expected with the values of |*V*_*cs*_| and |*V*_*cd*_| given by the CKMfitter group to within 2σ [36]. The error on the ratio |*V*_*cd*_/*V*_*cs*_|^2^ is currently dominated by the limited experimental statistics, and with the planned BESIII final data sample, we expect that the statistical uncertainty will be comparable to the systematic uncertainty that arises mainly from the LQCD decay constant calculations.

The matrix elements |*V*_*cs*_| and |*V*_*cd*_| can also be determined from the measured partial widths for the semi-leptonic decays $D^{0(+)} \rightarrow \bar{K} \ell \nu _\ell$ and *D*^0(+)^ → πℓν_ℓ_ with the computed values of the form factors from LQCD taken as inputs [28]. The results using this method are also shown in Fig. 7. At present, the uncertainties from LQCD calculations are 2.4% for $f^K_+(0)$ and 4.4% for $f^\pi _+(0)$, which are significantly larger than the uncertainties from the associated experimental measurements, and, therefore, limit the determinations of |*V*_*cs*_| and |*V*_*cd*_| with this approach.

**Figure 8.**
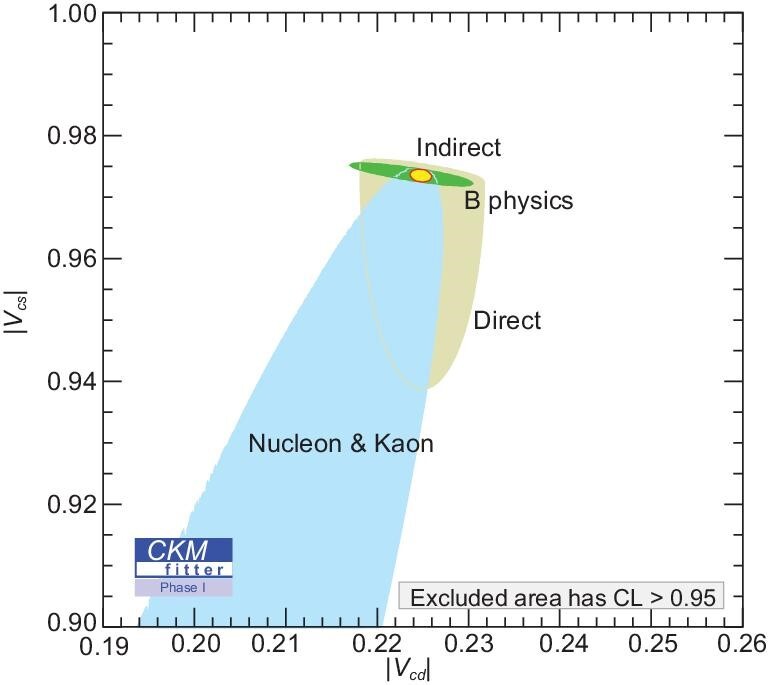
Constraints on the (|*V*_*cs*_|, |*V*_*cd*_|) plane expected with the future BESIII data-taking plan as described in the BESIII white paper [9]. The indirect (green) constraints (from *B* decays) are related to |*V*_*cs*_| and |*V*_*cd*_| by unitarity [36]. The direct (gray) constraints combine purely leptonic and semi-leptonic *D*_(*s*)_ decays from the BESIII experiment. The red circled region of the global combination corresponds to the 68% confidence level. Plot is from [9].

With the future BESIII data and improvements in the LQCD calculations on the decay constants and form factors that are expected circa 2025, we can anticipate significantly improved constraints on the (|*V*_*cs*_|, |*V*_*cd*_|) plane as shown in Fig. 8 [9], where direct contributions from the BESIII experiment are indicated. This will allow for precise tests of the consistency of CKM determinations from different quark sectors [9,36].

### Impact on the charm lifetime and SU(3)_F_ symmetry from inclusive semi-leptonic decays

Isospin symmetry requires that the charged and neutral *D* mesons have the same inclusive semi-leptonic partial widths for Cabibbo-favored decays [37], and this is confirmed by experiments within measurement uncertainties [21]. This prediction is expected to be reliable, since the lepton cannot interact strongly with the final-state hadrons, and the charged and neutral *D* mesons differ only in the isospin of the light quark. Therefore, a precise measurement of the Γ(*D*^0^ → *Xe*^+^ν_*e*_)/Γ(*D*^+^ → *Xe*^+^ν_*e*_) ratio (*X* refers to any accessible hadronic system) provides a test of isospin symmetry. With current values from the PDG [21], one has Γ(*D*^0^ → *Xe*^+^ν_*e*_) = (1.583 ± 0.027) × 10^11^ s^−1^ and Γ(*D*^+^ → *Xe*^+^ν_*e*_) = (1.545 ± 0.031) × 10^11^ s^−1^, and the observed ratio is

(7) \begin{equation*} R^{D^0/D^+}_{Xe\nu }=\frac{ \Gamma (D^0 \rightarrow X e^+ \nu _e)}{\Gamma (D^+ \rightarrow X e^+ \nu _e)} =1.025\pm 0.027. \end{equation*}

It indicates the need for reduction of experimental uncertainties on the branching fraction measurements before the predicted deviations of this ratio from unity can be identified. On the other hand, assuming the equality of semi-leptonic *D*^0^ and *D*^+^ partial widths, one obtains

$$\begin{eqnarray*}
&&\frac{\tau _{D^+}}{\tau _{D^0}} = \frac{\Gamma (D^0 \rightarrow \mathrm{all})}{\Gamma (D^+ \rightarrow \mathrm{all} )} = \frac{ \Gamma (D^0 \rightarrow \mathrm{all})}{\Gamma (D^0 \rightarrow X e^+ \nu _e)}\nonumber\\
&&\quad\qquad\times\, \frac{\Gamma (D^+ \rightarrow X e^+ \nu _e)}{\Gamma (D^+ \rightarrow \mathrm{all} )} = \frac{\mathcal {B}^{D^+}_{\rm SL}}{\mathcal {B}^{D^0}_{\rm SL}} ,
\end{eqnarray*}$$

where $\mathcal {B}^{D^+}_{\rm SL}$($\mathcal {B}^{D^0}_{\rm SL}$) is the inclusive semi-leptonic branching fraction for *D*^+^ (*D*^0^). Therefore, comparison of $\mathcal {B}^{D^+}_{\rm SL}/ \mathcal {B}^{D^0}_{\rm SL}$ with $\tau _{D^+}/\tau _{D^0}$ from direct lifetime measurements from other experiments provides a test of isospin symmetry in charm decays and QCD calculations. This analysis is currently ongoing at BESIII with triple the amount of CLEO-c data, and the sensitivity will be significantly improved.

Furthermore, inclusive semi-leptonic width measurements of strange and non-strange *D* mesons have revealed a clean determination of SU(3) breaking effects. According to the operator product expansion methods [38], the partial widths for the inclusive semi-leptonic decays of the *D*^+^, *D*^0^ and $D^+_s$ mesons should be equal up to SU(3)_F_ symmetry breaking and non-factorizable contributions (although their phase-space differences may not be trivial [38]). With the current values from the PDG [21], one has $\Gamma (D^+_s \rightarrow X e^+ \nu _e) = (1.300 \pm 0.082 ) \times 10^{11}$ s^−1^, and the observed ratio is

(8) \begin{equation*} R^{D_s/D}_{Xe\nu } = \frac{ \Gamma (D^+_s \rightarrow X e^+ \nu _e)}{\Gamma (D \rightarrow X e^+ \nu _e)} =0.830\pm 0.053. \end{equation*}

Thus, the semi-leptonic $D^+_s$ decay rate is $(17.0\pm 5.3)\%$ lower than the charge-averaged non-strange *D* semi-leptonic rate. This difference not only sheds light on strong-interaction dynamics, but can serve as a useful calibration for measurements using $D^+_s$ decays [38]. Inclusive $D^+_s \rightarrow X e^+ \nu _e$ decay is currently being studied at BESIII with an expected precision that will be comparable to that achieved for the corresponding *D* → *Xe*^+^ν_*e*_ mode.

Similar to the cases for the charmed mesons (*D*^0^/*D*^+^/$D^+_s$), the lifetime of the $\Lambda ^+_c$ charmed baryon is dominated by the weak decay of the charm quark, but is somewhat affected by the influence of the two accompanying light quarks (*u* and *d*) in the hadron state in contrast to the single light quark component in the meson case. Therefore, it will be interesting to make a comparison between the partial widths of the inclusive semi-leptonic decays $\Lambda ^+_c \rightarrow X e^+ \nu _e$ and *D* → *Xe*^+^ν_*e*_, so that one can further understand the internal interactions and structures in the charmed baryon and mesons. Information about exclusive semi-leptonic decays of the $\Lambda ^+_c$ is sparse [21], and only the $\Lambda ^+_c \rightarrow \Lambda \ell ^+ \nu _\ell$ (ℓ = *e* and μ) decay mode has been measured. The measurement of the branching fraction of $\Lambda ^+_c \rightarrow \Lambda \ell ^+ \nu _\ell$ was first performed by the ARGUS collaboration [39] and subsequently by the CLEO collaboration [40]. Recently, the BESIII collaboration measured the absolute branching fraction of $\Lambda ^+_c \rightarrow \Lambda \ell ^+ \nu _\ell$, as discussed in the section entitled ‘Precision measurements of the transition form factors’ [33,34]. A comparison of the exclusive semi-leptonic decay and the inclusive semi-leptonic decay will guide searches for new semi-leptonic decay modes. Based on the thres-hold data at BESIII, the absolute branching fraction of the inclusive semi-leptonic decays of the $\Lambda ^+_c$ baryon is determined to be $\mathcal {B}(\Lambda ^+_c \rightarrow X e^+ \nu _e)= (3.95\pm 0.34_\mathrm{stat} \pm 0.09_\mathrm{syst})\%$ [41], from which we obtain [41]

$$\begin{eqnarray*}
&&\mathcal {B}(\Lambda ^+_c \rightarrow \Lambda e^+ \nu _e)/ \mathcal {B}(\Lambda ^+_c \rightarrow X e^+ \nu _e)\\
&&\qquad = (91.9\pm 12.5_\mathrm{stat} \pm 5.4_\mathrm{syst})\%
\end{eqnarray*}$$

and determine the ratio

$$\begin{eqnarray*}
&&\Gamma (\Lambda ^+_c \rightarrow X e^+ \nu _e) / \Gamma (D\rightarrow X e^+ \nu _e)\\
&&\qquad = 1.26\pm 0.12,
\end{eqnarray*}$$

which can be used to restrict different QCD models and understand the internal interactions and structures in the charmed baryon and mesons [38,42,43].

## UNIQUE PROBES WITH QUANTUM ENTANGLED ${\boldsymbol {D^0\bar{D}^0}}$ AND ${\boldsymbol{ \Lambda _c^+ \bar{\Lambda }_c^-}}$ STATES

BESIII operating at the ψ(3770) resonance is a ‘charm factory’ that produces $D^0\bar{D}^0$ pairs in a state of definite charge-conjugation eigenvalue *C* = −. The antisymmetry of the wave function of the $D^0\bar{D}^0$ state induces quantum entanglement between the decay amplitudes of two *D* mesons. In particular, if one *D* meson is reconstructed in a *CP* eigenstate, the other *D* meson is required to have the opposite *CP* quantum number, provided *CP* is conserved in *D* decays. Thus, the transition $\psi (3770) \rightarrow D^0\bar{D}^0$ occupies a special place in the charm experimentalist’s and theorist’s arsenal [44]. BESIII data at ψ(3770) offer crucial experimental advantages for the determination of absolute branching fractions and interference between the two decay amplitudes from the entangled *D*^0^ and $\bar{D}^0$ mesons [45–48], that can be used to access their relative strong phases [12]. This suite of measurements is important to the international program in precision flavor physics and widely considered to be one of the main motivations for a charm factory [9]. Particularly pertinent to this review, BESIII offers unique opportunities to search for *CP* violation by exploiting quantum coherence in an almost background-free environment.

Analogously, the reaction $e^+ e^- \rightarrow \Lambda ^+_c \bar{\Lambda }^-_c$ produces charmed and anti-charmed baryon pairs. The $\Lambda ^+_c \bar{\Lambda }^-_c$ pair must be in a *C*-odd quantum entangled state, which provides a unique opportunity to study the spin observables in the charmed baryon decays at BESIII.

In this section, we discuss some selected measurements, including: (1) the unique quantum-coherent measurement of strong phases in neutral *D*^0^ hadronic decays and (2) the absolute branching fraction measurements of the $\Lambda _c^+$ hadronic decays firstly implemented in the cleanest way near thres-hold. Both of these topics are highlight results in charm physics at BESIII.

### Relative strong phase and constraints on the *CP*-violation phase **γ**

The complex phases that result from the strong interactions between the hadrons in the final states cannot be reliably calculated in theory and must be determined experimentally [12]. The values of the strong phase differences between the Cabibbo-favored (CF) and doubly Cabbibo-suppressed (DCS) amplitudes in charmed meson decays are crucial inputs for the extraction of the *CP*-violation phase angle γ, i.e. the phase of the CKM matrix element *V*_*ub*_ [16,49], determined from measurements of *b*-hadron decays. A precise measurement of γ provides a benchmark for tests of the SM that can be used as a probe to search for evidence of physics beyond the SM [36,50,51]. Three methods have so far been proposed to determine γ: GLW [52,53], ADS [54,55] and Dalitz (GGSZ) [56] analyses. One of the most sensitive decay modes for measuring γ is *B*^−^ → *DK*^−^ with *D* → *K*_*S*_π^+^π^−^ [56], where *D* represents a superposition of *D*^0^ and $\bar{D}^0$ mesons. The model-independent approach [57,58] requires a binned Dalitz plot analysis of the amplitude-weighted average cosine and sine of the relative strong phase between *D*^0^ and $\bar{D}^0\rightarrow K_S \pi ^+\pi ^-$ decay amplitudes to determine γ. These relative strong phases can be uniquely determined from quantum-correlated $\psi (3770) \rightarrow D^0\bar{D}^0$ decays.

In 2009 and 2010, the CLEO experiment presented first measurements of the strong-phase parameters by using 0.82 fb^−1^ of data [59,60]. The limited precision of these strong phase parameters translates into a systematic uncertainty for the measurement of γ of approximately 4° [61]. In the coming decade, the statistical uncertainty of γ is expected to be 1.5° or less, in which case the overall precision will be limited by the strong-phase inputs. Hence, measurements of the relative strong-phase parameters with improved precision are a high priority activity that is critical for a range of *CP*-phase measurements. At present, BESIII is the only running experiment that can take data at the $D^0\bar{D}^0$-pair-production threshold. Based on 2.93 fb^−1^ of data, BESIII recently explored several methods to improve the analysis by incorporating more hadronic *D*^0^ decays to increase statistics, including the development of partial reconstruction techniques to improve signal efficiency, and taking the effects of bin migration into account to reduce possible deviations of the results [62–64]. These improvements are critical to provide better precision and accuracy compared to previous measurements [61]. Using the new BESIII results, the effect of the strong-phase uncertainty on the value of γ will be reduced to around 1.0°, which is approximately a factor of 3 smaller than what was possible with the CLEO measurements [59,60]. This will ensure that the anticipated statistical improvements in the measurements of *CP*-phase γ at the LHCb and Belle II experiments over the next decade will have a deep impact on precision tests of the SM.

**Figure 9.**
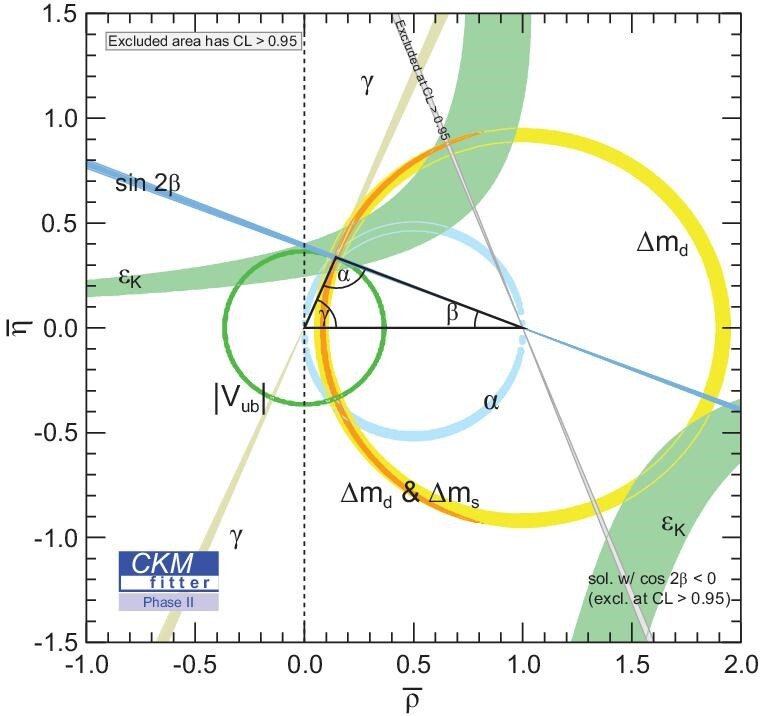
Evolving constraints and the global fit in the $(\bar{\rho }, \bar{\eta })$ plane (for $\bar{\rho }$ and $\bar{\eta }$, see Equation (2)) with the anticipated improvements by considering the LHCb upgrade [1] and final data set from the Belle II experiment [65]. The *CP*-violation phase γ is expected to have an accuracy around 0.4°, with the input from 20 fb^−1^ of data accumulated at the $D\bar{D}$ threshold [9] at BESIII. The shaded areas have the 95% confidence level. The plot is from the CKM fitter group [36] for the BESIII future physics programme [9]. Plot is from [9].

Given the future sensitivities resulting from the LHCb upgrade [1] and the final data set from Belle II [65], the overall constraints and the global CKM fit on the $(\bar{\rho }, \bar{\eta })$ plane (for $\bar{\rho }$ and $\bar{\eta }$, see Equation (2)) are shown in Fig. 9. The *CP*-phase γ is expected to have an accuracy of around 0.4° [66], thanks to the BESIII charm input that will be available with the full data sample of 20 fb^−1^ at the $D\bar{D}$ mass threshold that is part of the experiment’s future running plan [9]. Based on the future largest $D\bar{D}$ sample, quantum-coherence measurements of strong phases of more charm decay modes, as stated in [9], facilitate stringent cross checks of independent approaches of determining the γ angle and provide constraints in worldwide averaging the *D*^0^–$\bar{D}^0$ mixing parameters and the involved indirect *CP* violation. The future high-statistics *B* decay data at future LHCb upgrade provide sensitivity in accessing the strong phase parameters in principle. However, the final BESIII measurement is a necessary input for reaching the target γ sensitivity.

### Absolute branching fraction measurements of the **Λ_*c*_** decays

Measurements of weak decays of charmed baryons provide useful information for understanding the interplay of weak and strong interactions, and are complementary to the information obtained from charmed mesons. The lightest charmed baryon $\Lambda _{c}^{+}$, with quark configuration *udc*, serves as the cornerstone of charmed baryon spectroscopy. However, the progress of the theoretical understanding of $\Lambda _{c}^{+}$ decays has been slow [51,67–73], mostly due to limited understanding of the non-perturbative effects in QCD theory in the charmed baryon sector.

Before 2014, most $\Lambda _{c}^{+}$ decay branching fractions were obtained by measuring their ratios to the reference mode $\Lambda _{c}^{+}\rightarrow p K^-\pi ^+$, thus introducing strong correlations and compounding uncertainties. The old, experimentally averaged branching fraction, $\mathcal {B}({\Lambda _{c}^{+}\rightarrow p K^-\pi ^+})=(5.0\pm 1.3)\%$, had a large uncertainty due to the introduction of model assumptions on $\Lambda _{c}^{+}$ inclusive decays in these measurements [74]. Furthermore, only about 40% of the total decay rate had been measured and many modes were not identified, such as those with final-state neutrons. Therefore, comprehensive experimental measurements of various $\Lambda _{c}^{+}$ hadronic decays play an important role in improving different theoretical calculations [75] and developing the QCD methodology in handling non-perturbative effects.

Based on a 567 pb^−1^ data sample accumulated at 4.6 GeV, BESIII has systematically investigated the production and decays of the $\Lambda _c^+$ [9] for the first time using near-threshold data, which guarantee clean background and controllable systematics. BESIII provided absolute measurement of $\mathcal {B}{(\Lambda _{c}^{+}\rightarrow p K^-\pi ^+)}$ by counting the relative yields of the detected $\Lambda _c^+\bar{\Lambda }{}_c^-$ pairs over the single $\Lambda _c^+$, with the result (5.84 ± 0.27_stat_ ± 0.23_syst_)% [76]. This has competitive precision to the result ($6.84\pm 0.24^{+0.21}_{-0.27}$)% reported by Belle [77] at nearly the same time, and the combined precision of the two measurements is 5.2%, a five-fold reduction of the previous uncertainty [78]. Since this mode is the golden channel for detecting $\Lambda _c^+$ baryons in hadron collider experiments, the BESIII result impacts many aspects of heavy flavor physics. For instance, since the $\Lambda _b^0$ decays primarily to $\Lambda _{c}^{+}$ [42,79], it constrains the measurement of |*V*_*ub*_| via $\Lambda _b^0\rightarrow \Lambda _{c}^{+}\mu ^- \nu$. Improved measurements of $\Lambda _{c}^{+}$ hadronic decays can be used to constrain charm and bottom quark fragmentation functions by counting inclusive heavy flavor baryons [80].

In addition, BESIII reported numerous absolute branching fraction measurements of two-body CF and singly Cabibbo-suppressed (SCS) decays with improved precision, as listed in Table 3. The calculated branching fractions for these modes still have large uncertainties, and precise experimental measurements are essential to calibrate different models and explore the dynamics in charmed baryon decays. For instance, the improved precision provides crucial input to the theoretical predictions [88] for the observation channels of the doubly charmed baryon $\Xi _{cc}^{++}$ at LHCb [89]. In particular, the improved precision of the SCS modes is useful for testing SU(3)_F_ symmetry in the charm sector and provides insight of the size of *CP* violation in the charmed baryon sector [90].

**Table 3.** Measurements of the $\Lambda _c^+$ hadronic decays (two-body CF, neutron-involved and SCS decays) at BESIII, and their comparisons to the previous world averages. For BESIII results, the first uncertainties are statistical and the second are systematic.

		Previous world
Decay channel	BESIII (%)	averages (%) [78]
**Two-body CF**		
$pK^0_{S}$	1.52 ± 0.08 ± 0.03 [76]	1.15 ± 0.30
Λπ^+^	1.24 ± 0.07 ± 0.03 [76]	1.07 ± 0.28
Σ^0^π^+^	1.27 ± 0.08 ± 0.03 [76]	1.05 ± 0.28
Σ^+^π^0^	1.18 ± 0.10 ± 0.03 [76]	1.00 ± 0.34
Σ^+^ω	1.56 ± 0.20 ± 0.07 [76]	2.7 ± 1.0
Ξ^0^*K*^+^	0.590 ± 0.086 ± 0.039 [81]	0.50 ± 0.12
Ξ(1530)^0^*K*^+^	0.502 ± 0.099 ± 0.031 [81]	0.4 ± 0.1
Σ^+^η	0.41 ± 0.19 ± 0.05 [82]	0.70 ± 0.23
Σ^+^η^′^	1.34 ± 0.53 ± 0.19 [82]	First evidence
Σ(1385)^+^η	0.91 ± 0.18 ± 0.09 [83]	1.22 ± 0.37
**Neutron-involved**		
$nK^0_{S}\pi ^+$	1.82 ± 0.23 ± 0.11 [84]	First observation
Σ^−^π^+^π^+^	1.81 ± 0.17 ± 0.09 [85]	2.1 ± 0.4
Σ^−^π^+^π^+^π^0^	2.11 ± 0.33 ± 0.14 [85]	First observation
**SCS**		
*p*φ	0.106 ± 0.019 ± 0.014 [86]	0.082 ± 0.027
*p*η	0.124 ± 0.028 ± 0.010 [87]	First evidence
*p*π^0^	$<\!0.027\rm {at 90\%~C.L.}$ [87]	First measurement
*p*π^+^π^−^	0.391 ± 0.028 ± 0.039 [86]	0.35 ± 0.2
*pK* ^+^ *K* ^−^ (non-φ)	0.0547 ± 0.0130 ± 0.0074 [86]	0.035 ± 0.017

Moreover, BESIII observed, for the first time, decay modes with a neutron in the final state, including $\Lambda _c^+\rightarrow nK^0_{S}\pi ^+$ [84] and Σ^−^π^+^π^+^π^0^ with Σ^−^ → *n*π^−^ [85]. These analyses were carried out by using the missing-mass technique to infer the presence of a final-state neutron that is only possible because of the kinematic constrains of pair production in near-threshold data at BESIII. The results provide useful input to tests of isospin symmetry in the charm sector.

The hadronic weak decays of charmed baryons are expected to violate parity conservation. For instance, in a two-body decay $\Lambda _c^+\rightarrow BP$ (*B* denotes a $J^P =\frac{1}{2}^+$ baryon and *P* denotes a *J*^*P*^ = 0^−^ pseudoscalar meson) the parity asymmetry is defined as $\alpha ^+_{BP}\equiv {2{\rm Re}(s^* p)}/{(|s|^2+|p|^2)}$, where *s* and *p* stand for the parity-violating *s*-wave and parity-conserving *p*-wave amplitudes in the decay, respectively. For the process $\Lambda _c^+\rightarrow \Lambda \pi ^+$, which proceeds via a *W* interaction, *c* → *W*^+^ + *s*, the effects of parity violation are mainly determined by studying the polarization of the produced Λ via its decays to *p*π^−^ from the initially (polarized) charmed baryons [75,91]. If *CP* is violated, the decay asymmetry parameters $\alpha ^+_{BP}$ for $\Lambda _{c}^{+}$ and $\bar{\alpha }^-_{\bar{B}\bar{P}}$ for $\bar{\Lambda }{}_{c}^{-}$ have different magnitudes but are opposite in sign. Hence, separate determinations of $\alpha ^+_{BP}$ and $\bar{\alpha }^-_{\bar{B}\bar{P}}$ would facilitate searching for the effects of *CP* violation. So far, only a few decay asymmetry parameters, e.g. α_Λπ_ for $\Lambda _c^+\rightarrow \Lambda \pi ^+$, and $\alpha _{\Sigma ^+\pi ^0}$ for $\Lambda _c^+\rightarrow \Sigma ^+\pi ^0$, have been studied, and even those with limited precision [21]. Therefore, improved or new decay asymmetry measurements are desirable, as they could shed light on the decay mechanism and allow searches for *CP* asymmetries in the charmed baryon sector. In addition the decay asymmetry values allow for discrimination between different theoretical models, as listed in [75,92].

**Figure 10.**
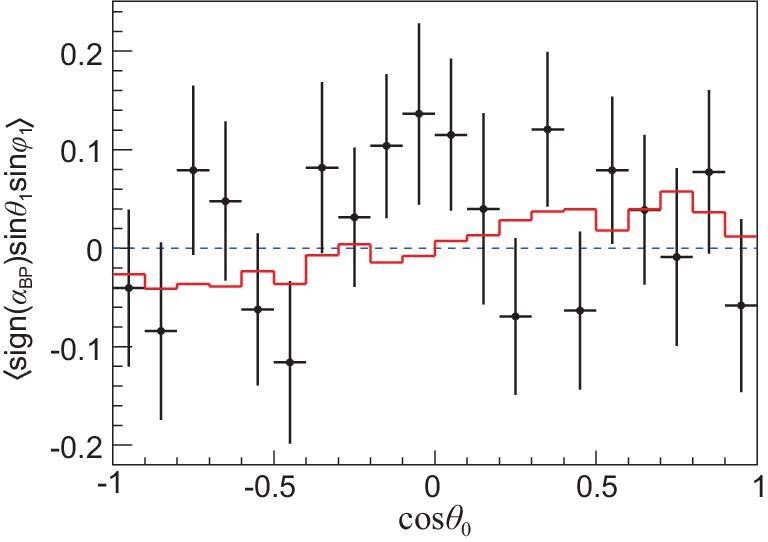
The effect of the $\Lambda _c^+$ transverse polarization versus cos θ_0_ in $e^+e^- \rightarrow \Lambda _c \bar{\Lambda }_c$ at a center-of-mass energy of 4.6 GeV. Here θ_0_ is the $\Lambda _c^+$ production angle relative to the *e*^−^-beam direction. The solid curve is a fit to the data; the dotted line is the expectation for zero polarization. Detailed description can be found in [93]. Plot is from [9].

In the near-threshold production of $\Lambda _c^+\bar{\Lambda }_c^-$ pairs, non-zero transverse polarization of the $\Lambda _c^+$ will aid the determinations of the decay asymmetries. The decay asymmetry parameters are determined by analyzing the multi-dimensional angular distributions, where the full cascade decay chains are considered. The detailed method can be found in [93], in which a joint extraction of the four decay parameters of α_Λπ_, $\alpha _{\Sigma ^+\pi ^0}$, $\alpha _{\Sigma ^0\pi ^+}$ and $\alpha _{p\bar{K}^0}$ at the same time was carried out based on the $\Lambda _{c}^{+}$ sample at 4.6 GeV. An indiction of a non-zero transverse polarization is seen with a significance of 2.1σ, as shown in Fig. 10, which makes the measurement of the asymmetry parameter $\alpha _{p\bar{K}^0}$ accessible experimentally for the first time. The asymmetry parameters [93] for the $p\bar{K}^0$, Λπ^+^, Σ^+^π^0^ and Σ^0^π^+^ modes are measured to be 0.18 ± 0.43_stat_ ± 0.14_syst_, −0.80 ± 0.11_stat_ ± 0.02_syst_, −0.57 ± 0.10_stat_ ± 0.07_syst_ and −0.73 ± 0.17_stat_ ± 0.07_syst_, respectively. In comparison with previous results, the measurements for the Λπ^+^ and Σ^+^π^0^ modes are consistent but have an improved precision, while the parameters for the $p\bar{K}^0$ and Σ^0^π^+^ modes are measured for the first time. At present, no theoretical model provides predictions that are fully consistent with all these measurements and BESIII measurements have become benchmarks to calibrate the QCD-derived theoretical models. During BESIII’s 2020 data taking, about 10 times larger $\Lambda _c^{+}$ samples were accumulated at the center-of-mass energies between 4.6 and 4.7 GeV, and the significance of $\Lambda _c^+$ polarization could improve to more than 5σ. In this case the precision of the decay asymmetries will be improved at least by a factor of 3. With this information, tests of *CP* violations can be pursued for the two-body decays by comparing decay asymmetry parameters measured separately for $\Lambda _c^{+}$ and $\bar{\Lambda }_c^{-}$.

## SUMMARY AND PROSPECTS

Charm particle weak decays remain an exciting field for both theoretical and experimental investigations. In this article, we summarize results on charm decays that have been obtained in the BESIII experiment with data sets collected at the production thres-holds of $D\bar{D}$, $D_s^{*+} D^{-}_s$ and $\Lambda ^+_c \bar{\Lambda }^-_c$. These data samples allow the application of double-tag methods to fully reconstruct events even when invisible particles, such as neutrons or neutrinos, are present in the final states. This provides a unique environment to obtain the absolute branching fractions of charmed hadron decays to purely leptonic, semi-leptonic and hadronic final states with very low background levels. These BESIII measurements provide rigorous tests of QCD-based models and measurements of the CKM matrix elements |*V*_*cs*_| and |*V*_*cd*_|, supply inputs to CKM weak phase measurements and test leptonic-flavor universality.

Charmed hadron studies will continue during the future upgrade of the BESIII experiment. By the end of the BESIII program, which will include some important machine upgrades, 10 times the current amount of data will be collected [9], and this will usher in a precision charm flavor era. High statistics data near the production thresholds with quantum-coherent initial states at BESIII will provide key measurements of the phase differences between the decay amplitudes while no reliable QCD-based computation is available. This suite of measurements is important to the worldwide flavor physics program. New inputs from future BESIII analyses based on larger data samples will deepen our understanding of the detailed dynamics of charm decays and hopefully facilitate reliable theoretical predictions for the *CP* asymmetry in the charm sector [3], therefore allowing us to search for new physics beyond the SM.

In addition, other experiments, such as LHCb and Belle II, are running and will produce huge statistics of charm hadrons, providing stringent constraints on *CP*-violation observables [3]. The sensitivity of the observed *CP* asymmetry in charmed meson decays by LHCb is about 3 × 10^−4^ [2], which is consistent with the SM expectation $\mathcal {O}(10^{-4}-10^{-3})$ [94]. BESIII, with 20 fb^−1^ of data at the ψ(3770) peak, can only reach a sensitivity level of a few percent on the *CP*-violation measurements, and the corresponding sensitivity at a super-τ-charm factory [95,96] is only $\mathcal {O}(10^{-3})$, which is still one order of magnitude lower than that for the current LHCb data set [97]. However, a super-τ-charm factory has the potential to provide constraints on the decay dynamics of charmed hadrons [98]. All these experiments plus their future upgrades will continue the studies of charmed hadron physics that will deepen our understanding of strong interactions in the charm sector, and constrain the SM parameters. Finally, Table 4 presents the precision prospects for some key charmed hadron measurements that are based on the BESIII future data-taking plan.

**Table 4.** Prospects of some key measurements with the future data-taking plan in the BESIII white paper [9].

Observable	Measurement	BESIII [9]
$\mathcal {B}(D^+\rightarrow \ell ^+\nu _\ell )$	$f_{D^+}|V_{cd}|$	1.1%
$\mathcal {B}(D^+_s\rightarrow \ell ^+\nu _\ell )$	$f_{D^+_s}|V_{cs}|$	1.0%
${\rm d}\Gamma (D^{0/+}\rightarrow \bar{K}\ell ^+\nu _\ell )/{\rm d} q^2$	$f^K_{+}(0)|V_{cs}|$	0.5%
dΓ(*D*^0/ +^ → πℓ^+^ν_ℓ_)/d*q*^2^	$f^\pi _{+}(0)|V_{cd}|$	0.6%
${\rm d}\Gamma (D^+_s\rightarrow \eta \ell ^+\nu _\ell )/{\rm d} q^2$	$f^\eta _{+}(0)|V_{cs}|$	0.8%
Strong phases in *D*^0^	Constraint on γ	<0.4°
$\Lambda _c^+\rightarrow pK^-\pi ^+$	$\mathcal {B}(\Lambda _c^+\rightarrow pK^-\pi ^+)$	2%
$\Lambda _c^+\rightarrow \Lambda \ell ^+\nu _\ell$	$\mathcal {B}(\Lambda _c^+\rightarrow \Lambda \ell ^+\nu _\ell )$	3.3%
